# Validation of new marker of fluid responsiveness based on Doppler assessment of blood flow velocity in superior vena cava in mechanically ventilated pigs

**DOI:** 10.1186/s40635-018-0199-9

**Published:** 2018-09-24

**Authors:** Tomas Kovarnik, Miroslav Navratil, Jan Belohlavek, Mikulas Mlcek, Martin Chval, Zhi Chen, Stepan Jerabek, Otomar Kittnar, Ales Linhart

**Affiliations:** 1 2nd Department of Internal Medicine - Department of Cardiovascular Medicine, First Faculty of Medicine, Charles University in Prague and General University Hospital in Prague, Prague, Czech Republic; 2 Department of Physiology, 1st Faculty of Medicine, Charles University in Prague, Prague, Czech Republic; 3 Institute for Research and Development of Education, Faculty of Education, Charles University in Prague, Prague, Czech Republic; 4 Department of Electrical and Computer Engineering and Iowa Institute for Biomedical Imaging, The University of Iowa, Iowa City, IA USA; 5 II. interni klinika VFN a 1.LF UK, U nemocnice 2, 128 08 Praha 2, Czech Republic

**Keywords:** Fluid responsiveness, Hypovolemia, Hemodynamics, Flow measurement, Animal experiment, Superior vena cava

## Abstract

**Background:**

We studied a novel approach for the evaluation and management of volemia: minimally invasive monitoring of respiratory blood flow variations in the superior vena cava (SVC). We performed an experiment with 10 crossbred (Landrace × large white) female pigs (*Sus scrofa* domestica).

**Methods:**

Hypovolemia was induced by bleeding from a femoral artery, in six stages. This was followed by blood return and then an infusion of 1000 ml saline, resulting in hypervolemia. Flow in the SVC was measured by Flowire (Volcano corp., USA), located in a distal channel of a triple-lumen central venous catheter. The key parameters measured were venous return variation index (VRV)—a new index for fluid responsiveness, calculated from the maximal and minimal velocity time intervals during controlled ventilation—and systolic peak velocity (defined as peak velocity of a systolic wave using the final end-expiratory beat). A Swan–Ganz catheter (Edwards Lifesciences, USA) was introduced into the pulmonary artery to measure pulmonary arterial pressure, pulmonary capillary wedge pressure, and continuous cardiac output measurements, using the Vigilance monitor (Edwards Lifesciences, USA).

**Results:**

We analyzed 44 VRV index measurements during defined hemodynamic status events. The curves of VRV indexes for volume responders and volume non-responders intersected at a VRV value of 27, with 10% false negativity and 2% false positivity. We compared the accuracy of VRV and pulse pressure variations (PPV) for separation of fluid responders and fluid non-responders using receiver operating characteristic (ROC) curves. VRV was better (AUCROC 0.96) than PPV (AUCROC 0.85) for identification of fluid responders. The VRV index exhibited the highest relative change during both hypovolemia and hypervolemia, compared to standard hemodynamic measurement.

**Conclusions:**

The VRV index provides a real-time method for continuous assessment of fluid responsiveness. It combines the advantages of echocardiography-based methods with a direct and continuous assessment of right ventricular filling during mechanical ventilation.

## Background

Accurate detection of early hypovolemia remains a challenging issue for both perioperative monitoring and critical care units. It is essential for optimal volume therapy. Both unrecognized hypovolemia and fluid overload worsen a patient’s prognosis [1]. In routine clinical practice, we can use either static or dynamic markers for the detection of hypovolemia. These markers use well-known lung–heart interactions [2].

Static markers—such as central venous pressure (CVP), pulmonary capillary wedge pressure (PCw), urine output, heart rate, and blood pressure—are not sufficient to indicate early phases of hypovolemia. Furthermore, they do not reliably predict the response of cardiac output to volume therapy [3]. Dynamic markers—such as pulse pressure variation (PPV), systolic pressure variation (SPV), stroke volume variation (SVV), or a respiratory systolic variation test (RSVT)—better predict fluid responsiveness [4–6]. They are, however, highly affected by arrhythmias, tidal volume, and spontaneous breathing activity [7, 8]. Those related to peripheral arterial modalities frequently exhibit inherently false negativity in patients on vasopressor therapy. False positivity has been described in cases with pulmonary artery hypertension and/or compromised ventricular function [9, 10]. Dynamic markers more closely reflect lung–heart interaction, although they do not really determine volemia status. Rather, they indicate a patient’s position on the Frank–Starling curve [11] and therefore can predict fluid responsiveness [12]. Guyton et al. showed, in experiments performed on dogs in the mid-part of the last century, that cardiac output is equal to venous return (VR) [13]. The driving pressure for VR is mean systemic filling pressure, that is, the pressure to which all intravascular pressures—arterial and venous alike—equilibrate during conditions of zero flow (as in cardiac arrest) [12]. Because of this, some authors have studied flow changes in the superior vena cava (SVC) to better understand the filling of the heart from systemic venous return. These changes are real markers of right ventricle (RV) preload. The SVC collapsibility index has been shown to clearly differentiate fluid responders from non-responders in septic patients. This technique has been reliable, even in cases where pulse pressure variations were identified as false positive or false negative [14]. Unfortunately, Doppler analysis of SVC flow cannot be assessed with a transesophageal probe, due to the physical constrictions present in the angle between the esophagus and the SVC. The transthoracic approach for detection of SVC flow from the supraclavicular fossa has been well described in the literature [15, 16]. However, echocardiography-based measurement from the supraclavicular fossa requires an experienced echocardiographer, and these kinds of examinations are not suitable for continuous monitoring.

Based on the volume assessment limitations of the methods described, we propose a novel approach for the evaluation of intravascular volume status and fluid management. We hypothesize that minimally invasive monitoring of respiration-related blood flow variations—where they begin, in the SVC—might provide a superior fluid responsiveness index, especially when compared to measures derived from the left side of circulation. This would also be suitable for continuous measurement, unlike echocardiography-derived indices. To measure respiration-related variability in SVC flow, we used a wire with a miniaturized Doppler probe designed for intracoronary flow measurements (Flowire, Volcano corp., Rancho Cordova, CA, USA) incorporated in a central venous catheter.

## Methods

### Animals

Ten crossbred (Landrace × large white) healthy female pigs (*Sus scrofa* domestica), 4 to 5 months old, with a mean body weight of 51.6 ± 2.8 kg, were used in the study. The animals were obtained from a local certified farm, inspected on arrival by an institutional veterinarian, and were not subjected to any other interventions before or after the study protocol. Prior to the study, the animals were housed in a standard, accredited, university animal facility. They were kept in metal cages with minimum dimensions of 1.8 × 2.2 m, with natural daylight and free access to water, and were fed twice daily with a mixture recommended for young swine. Ambient conditions (temperature, air) were regulated according to relevant standards. Animals were kept in groups of up to four. Anesthesia was provided during the whole study.

### Anesthesia

After 24 h of fasting, sedation was induced by midazolam (0.3 mg/kg IM) followed by ketamine hydrochloride (15 to 20 mg/kg IM). Anesthesia was continued with initial boluses of propofol and morphine (2 mg/kg IV and 0.1 to 0.2 mg/kg IV, respectively), and animals were orotracheally intubated. A continuous IV infusion of propofol (8 to 10 mg/kg/h) combined with morphine (0.1 to 0.2 mg/kg/h) IV was used to maintain anesthesia, the depth of which was regularly assessed by photoreaction and corneal reflex.

### Ventilation

Volume-controlled ventilation was delivered by the Hamilton G5 ventilator (Hamilton Medical, Bondauz, Switzerland) set at Vt 8 ml/kg, PEEP 5 cm H_2_O, FiO_2_ 0.25, I:E 1:2, and MV adjusted to maintain pCO_2_ 5.0–5.5 kPa (34–41 mmHg) and pO_2_ 9.3–16 kPa (70–120 mmHg).

### Invasive measurements

A triple-lumen central venous oximetry catheter (Multi-Med, Edwards Lifesciences) was inserted via the left external jugular vein to allow for (i) insertion of the Doppler Flow wire (0.014″) via its distal lumen, (ii) central venous pressure measurement (CVP), and (iii) administration of i.v. medications.

A Swan–Ganz continuous cardiac output (CCO) catheter (Edwards Lifesciences, USA) was inserted via the left femoral vein and advanced into the pulmonary artery to provide measurements of pulmonary arterial pressure (PAP) and pulmonary capillary wedge pressure (PCWP). CCO and mixed venous saturation (S_V_O_2_) were measured using the Vigilance monitor (Edwards Lifesciences, USA). In addition, a high-fidelity pressure–volume catheter (7F VSL, connected to ADV 500 console, Transonic Scisense Inc., London, ON, Canada) was placed retrograde into the left ventricular apex via a 7F sheath inserted percutaneously into the left external carotid artery. The correct position was confirmed by fluoroscopy. This catheter provided simultaneous measurements of left ventricle (LV) pressure and volume. LV pressure–volume loops were obtained from PV data using Powerlab Pro 7.0. software (ADInstruments, USA).

An introducer sheath (5F) was inserted into the femoral artery for invasive arterial blood pressure measurement. Another 6F introducer was placed in the contralateral femoral artery for blood removal during the hypovolemia protocol. A further 7F sheath was inserted into the right femoral vein for rapid blood and fluid replacement.

All blood pressure measurements (excluding LV pressure) were obtained via a fluid-filled pressure transducer (TruWave, Edwards Lifesciences, USA) connected to a patient monitor (Lifescope TR, Nihon Kohden, Japan). Peripheral saturation, capnometry, and temperature recordings were also performed via the patient monitor.

### Doppler-based technique for superior vena cava blood flow assessment

The technical details of Doppler-based blood flow velocity measurement has been fully described previously, in a paper done by Doucette et al. [17]. A Flowire, connected to a Combomap console (Volcano Corp.), was inserted through the distal lumen of the central venous catheter, to the position in the SVC which had the best quality of laminar flow Doppler signal. The correct position was confirmed by fluoroscopy. The pulse Doppler of the SVC flow velocity was displayed on the console. The console automatically detected and plotted the envelope of the SVC flow velocity pattern. Data was read and recorded using the National Instruments LabView software. Doppler sampling frequency was 100 kHz.

The following parameters were recorded:

- SVC flow velocity as a velocity time integral (VTI) of the Doppler envelope.
- Venous return variation index (VRV), an analogue to the SVV as a new index for fluid responsiveness. We used airway pressure for respiration gating and ECG R-R intervals for heart beat detection. We analyzed flow variations during each individual respiratory cycle at 20-s intervals. Beats with maximal and minimal VTI values were used in the formula. Minimal flow occurred during mechanical inspiration; maximal flow was recorded in expiration. The VRV index was calculated as VRV = (VTImax − VTImin)/(VTImax + VTImin)/2. Individual respiratory cycle VRV values were averaged over a 20-s period.
- Systolic peak velocity (S peak) was also recorded. It is defined as peak velocity on a systolic wave using the final end-expiratory beat.

### Data recording

**Fig. 1:**
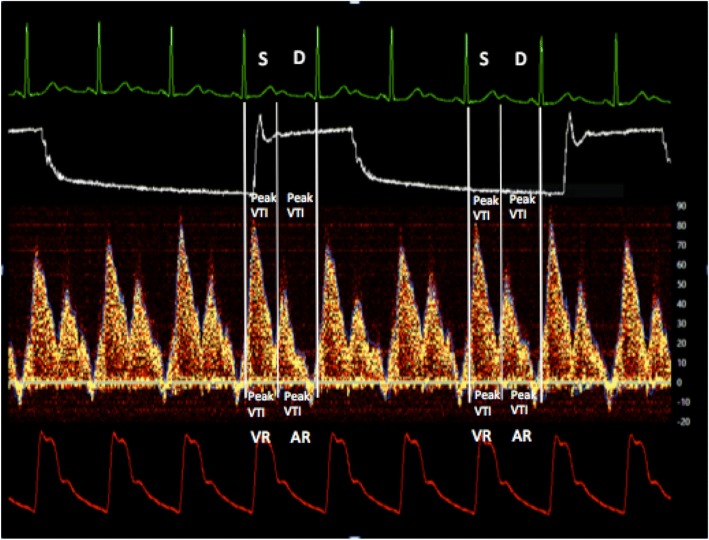
Screenshot from Doppler analyzer with ECG, airway pressures, flow in SVC, and blood pressure tracings. Shown are, from upper side to lower side, ECG, airway pressure, Doppler flow signal from the SVC, and arterial blood pressure. S systolic forward flow, D diastolic forward flow, VR systolic reversal flow, AR atrial reversal flow, VTI velocity time integral

Doppler envelope data were continuously recorded with a custom PC-based application specifically designed for the purposes of this trial. Other modalities recorded simultaneously by the same application were central venous pressure, arterial pressure, airway pressure, and ECG (Fig. 1). The application was designed to allow for both continuous manual and automatic parameter calculation (PPV, SVV, EDP, SVC flow with respiratory and ECG gating). Other physiological parameters were also recorded by the ADI LabChart Pro at 400 Hz (CCO, SvO_2_, PCWP, PAP, CVP, AP—arterial pressure, temperature).

### Medication

Initial rapid IV infusion of 1000 ml of normal saline was given intravenously, followed by a continuous IV infusion to reach and maintain a central venous pressure of between 3 and 7 mmHg.

Unfractionated heparin (100 UI/kg IV) was given as a bolus after sheath placement, followed by 40 to 50 U/kg/h continuous IV drip to maintain activated clotting time of 180 to 250 s (values were checked every hour with Hemochron Junior+, International Technidyne Corporation, Edison, NJ, USA).

### Study protocol

After sedation and ventilation set-up, the animals were kept in euvolemic status.

Euvolemia was defined as:

- An increase of stroke volume (SV) < 10% after saline bolus of 7 ml/kg
- A stroke volume respiratory variation < 15% (LV Volume with Powerlab Pro PV tools)

Euvolemia was then followed by bleeding periods.

The bleeding was induced by opening the 6F sheath introduced to the femoral artery. Blood was collected in transfusion sets, which were stored at room temperature. Estimated blood volume (EBV) was calculated as 65 ml/kg [18].

The first stage of bleeding removed 10% of EBV. This was followed by five subsequent stages, which each removed 5% of the initial EBV. A steady state of 10 min was kept after each bleeding period, after which measurements were performed.

The last bleeding was followed by two stages of 500 ml blood return (each lasting 30 min) separated by 10-min steady states. Fluid challenge (1000 ml saline over 30 min, given two times, separated by 10 min of steady state) administered after a non-responsive event was classified as fluid overload.

We were searching for a new predictor of fluid responsiveness, defined as an increase in the stroke volume by more than 10% (in comparison with the previous stage of the experiment).

#### Statistic analysis

The data presented is from 10 animals with a total of 44 events classifiable as responsive or non-responsive, in terms of changes in stroke volume variations. Fluid responsiveness was defined as a 10% change in stroke volume after all three study steps: bleeding, blood return, and fluid overload. During the trial, some events were not classifiable due to arrhythmias, PAC failure, or Doppler envelope detection failure. Five cases provided complete continuous trending data of all measured variables.

## Results

### VRV index as a marker of hemodynamic status

**Fig. 2:**
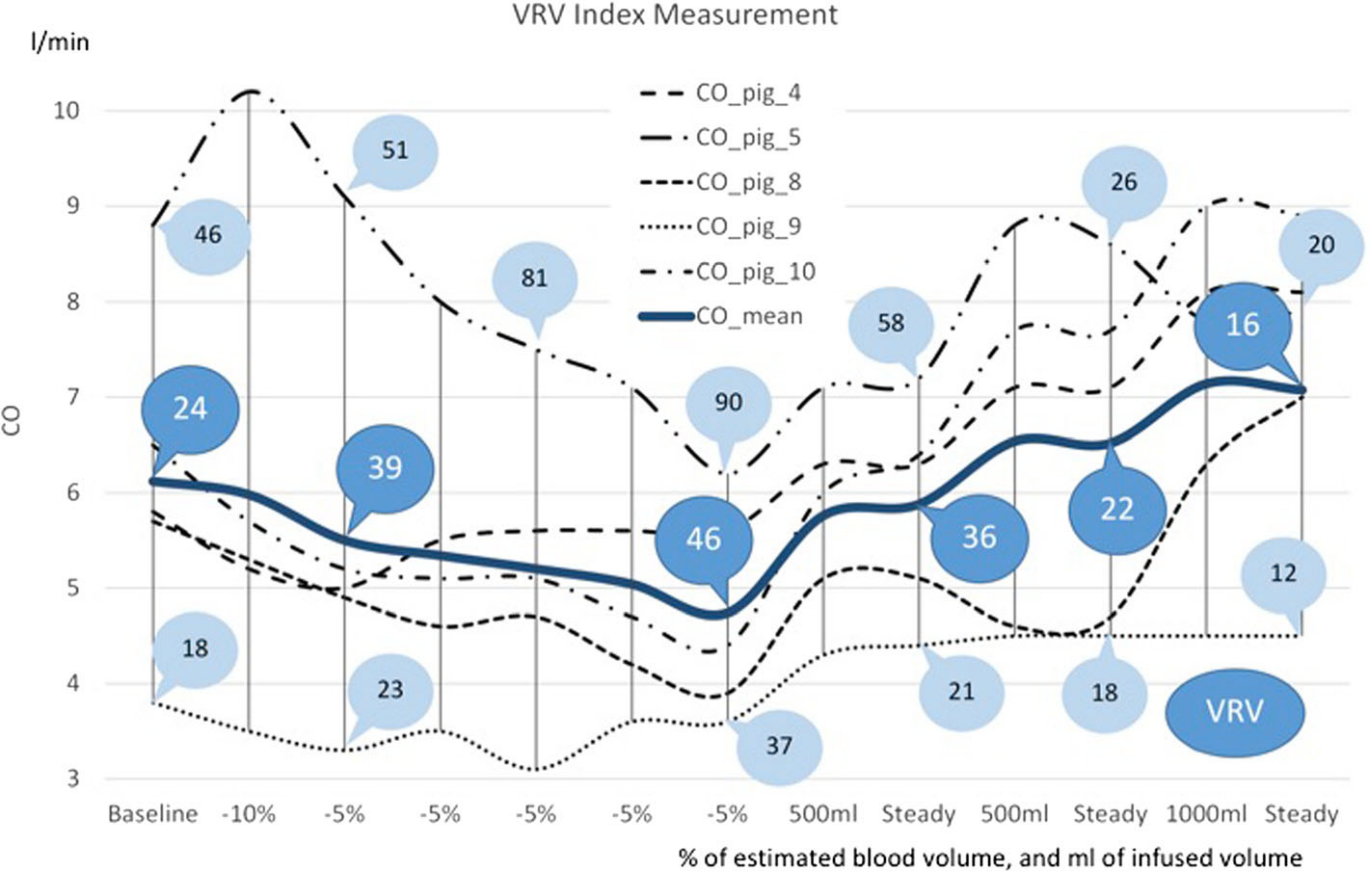
Changes in VRV index during the study. The “*X*” axis represents changes in volume status (bleeding in percent of estimated blood volume, two times reinfusion with 500 ml of blood, 1000 ml of saline infusion). The “*Y*” axis represents cardiac output in liters per minute measured by CCO (SwanGanz catheter). Numbers in circles are VRV indexes. This figure was done using data from five animals. VRV venous return variation index, CO cardiac output

**Fig. 3:**
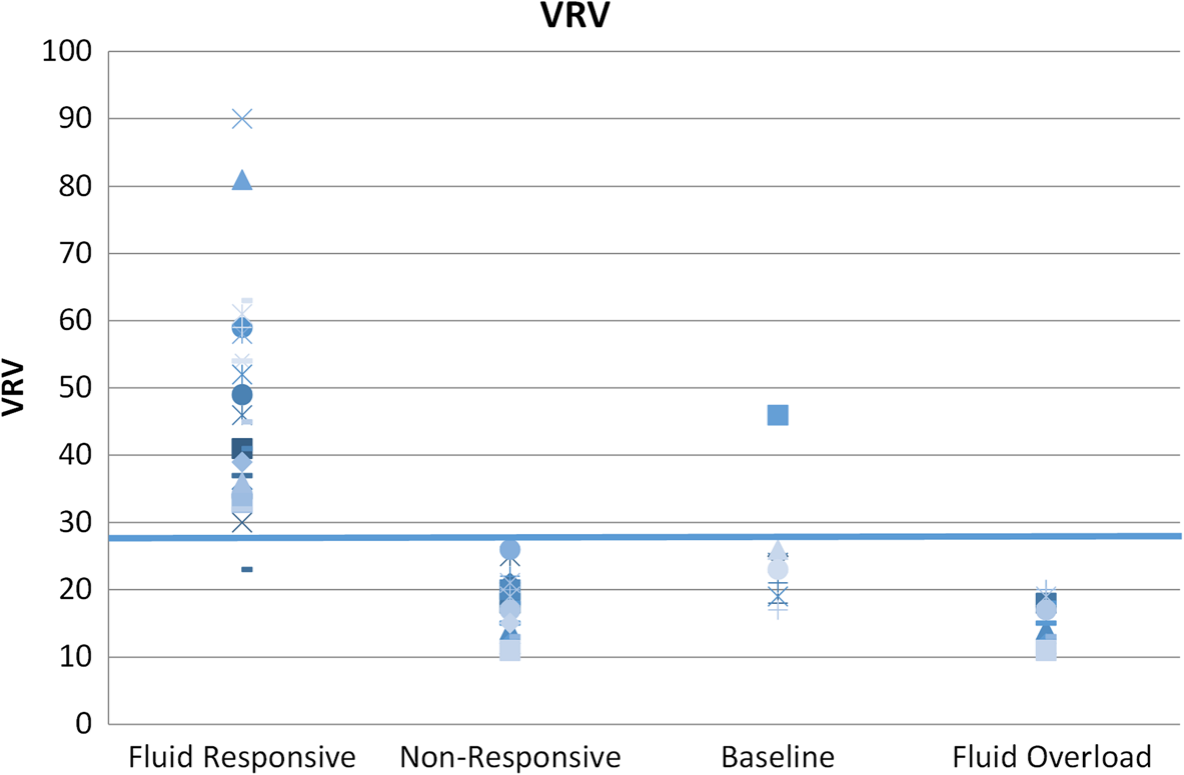
VRV index in all hemodynamic situations. VRV venous return variation index

**Fig. 4:**
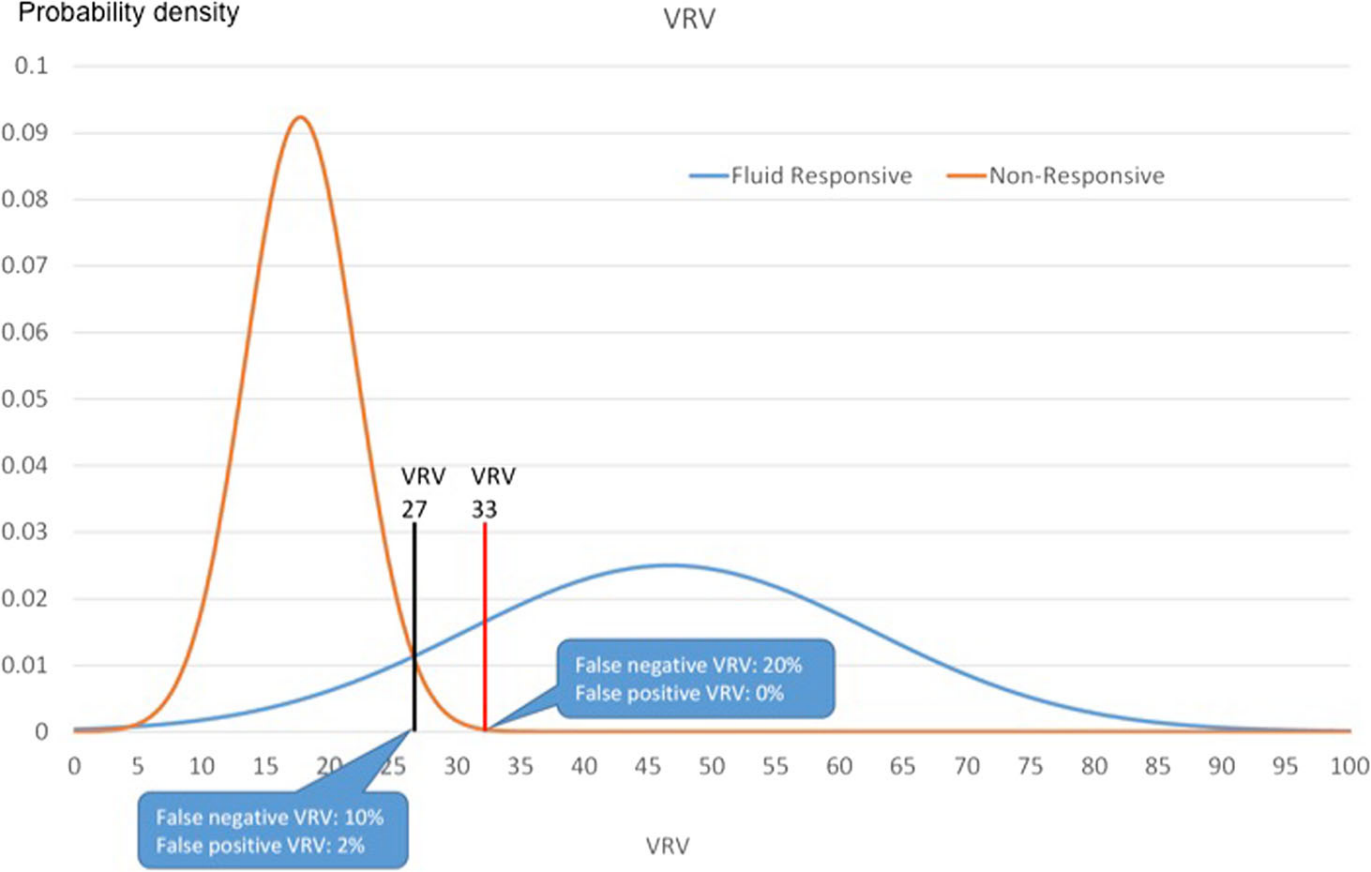
The curves of VRV indexes for volume responders and volume non-responders. VRV venous return variation index

The relationship between CO and VRV, during all phases of the experiment, is presented in Fig. 2. This represents data from five animals which completed all phases of the study protocol. VRV values from all 10 animals were dichotomously divided into fluid-responsive or fluid-non-responsive categories. These values are shown—together with two more categories (baseline values for VRV and VRV values during fluid overload)—in Fig. 3. Normal distributions of the VRV index were found for both categories of hemodynamic status (fluid-responsive or fluid-non-responsive) (Fig. 4).

**Fig. 5:**
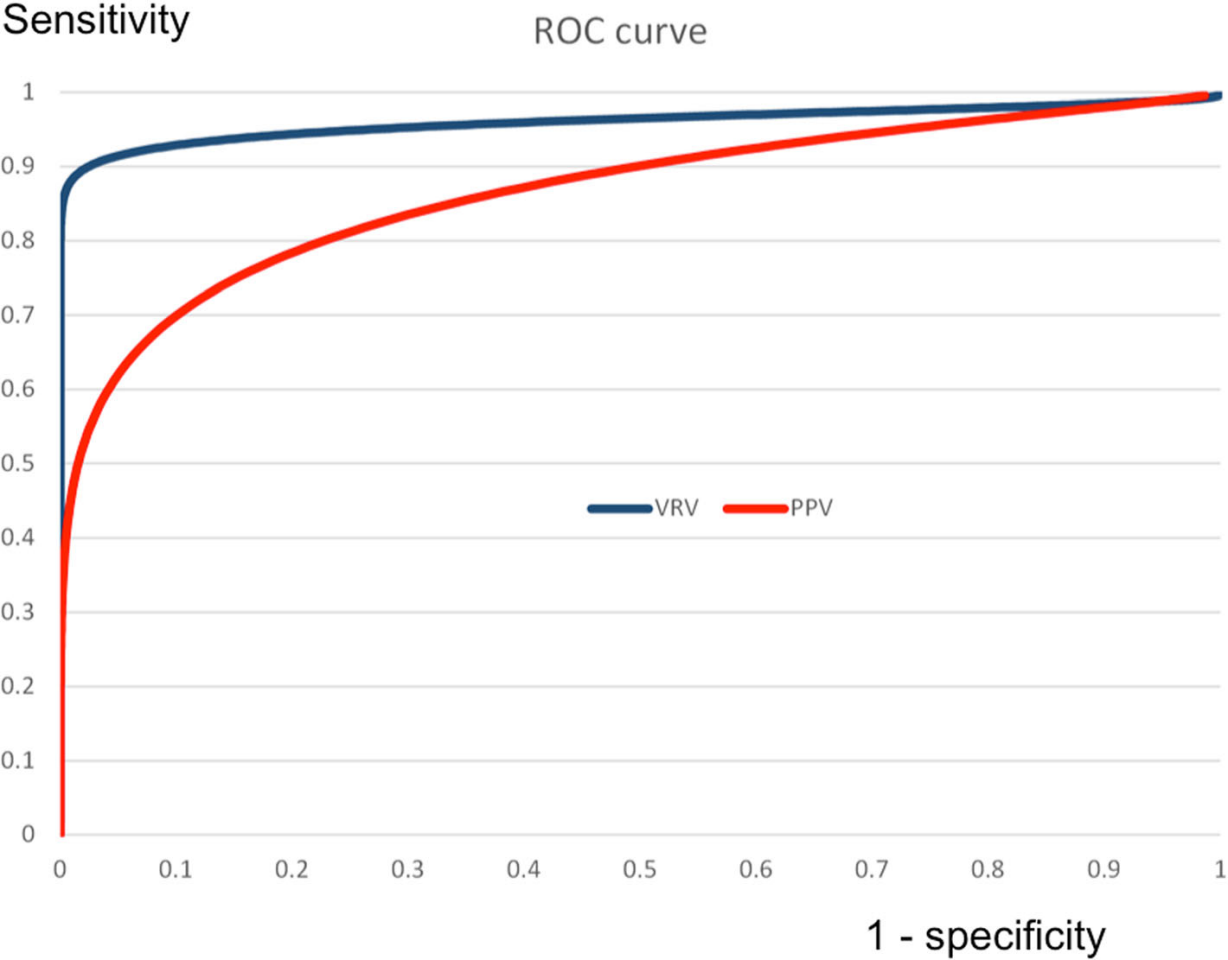
AUC curves for VRV (0.96) and PPV (0.85) for prediction of fluid responsiveness. AUC area under curve, VRV venous return variation index, PPV pulse pressure variations

We used a receiver operating characteristic curve (ROC), for comparison of the VRV and PPV indexes to predict fluid responsiveness (Fig. 5).

### VRV index as a tool for prediction of volume responsiveness

We were searching for a cutoff level for the VRV index (for detection of fluid responsiveness) from a total of 44 measurements of VRV during defined hemodynamic status. The curves of VRV indexes for volume responders and volume non-responders intersected at a VRV value of 27, with 10% false negativity and 2% false positivity. The upper limit of the gray zone—with a 100% prediction of fluid responsiveness—is at the VRV value of 33. At this point, the false negative rate would be at 19.6% (Figs. 3 and 4).

Based on these results, the VRV index could be a very useful tool for fluid management as follows:

- A VRV index value > 33 is a strong marker of fluid responsiveness. The risk of a false negative test for this value is 20%.
- A VRV index value of 27 was a good predictor of fluid responsiveness, with a risk for false negative results of 10% and for false positive results of 2%.
- A VRV value < 20 was a marker for hypervolemia.

### VRV index versus PPV as the markers of fluid responsiveness

**Fig. 6:**
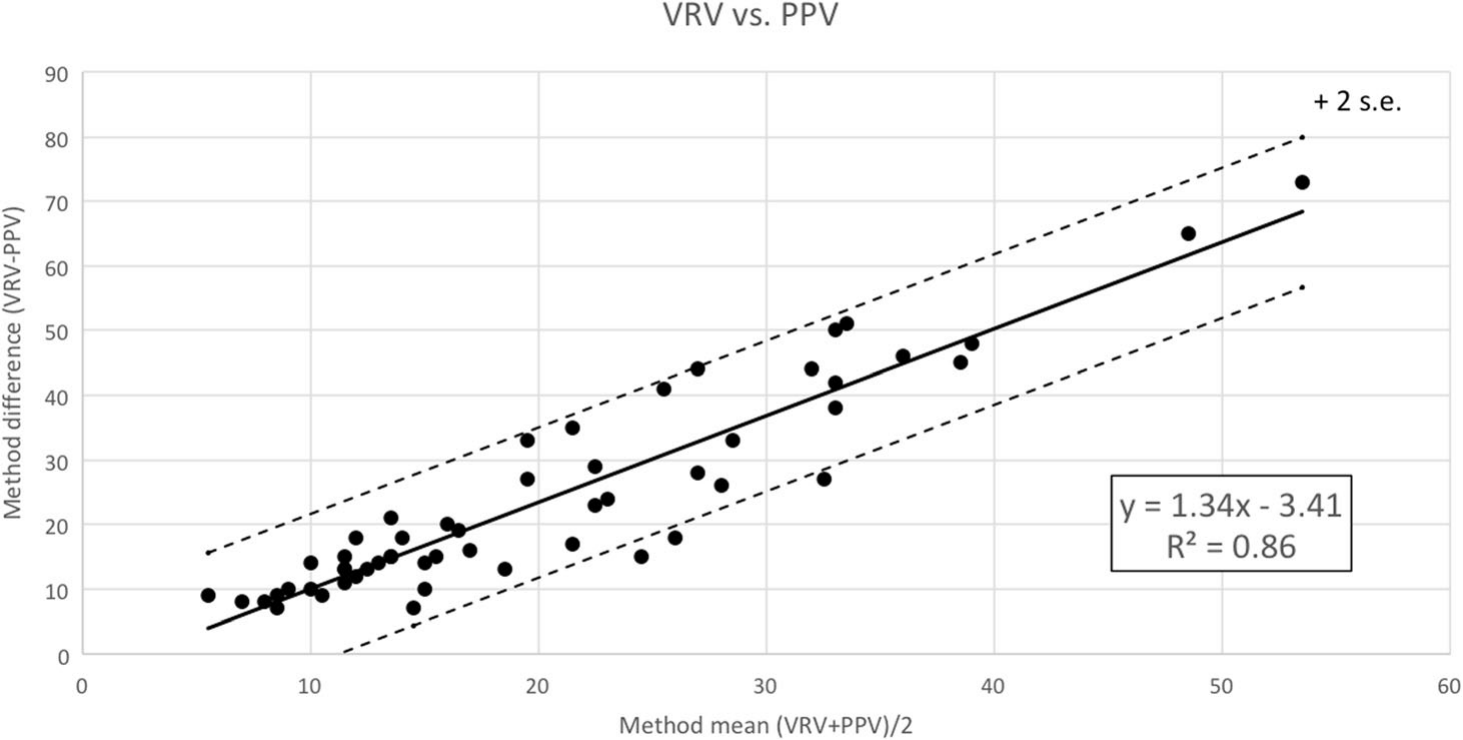
Bland–Altman plots on agreement measures between VRV and PPV. It shows the biggest difference between VRV and PPV around the VRV cutoff point for fluid responsiveness. VRV venous return variation index, PPV pulse pressure variations

PPV is calculated as 100 × [(PPmax − PPmin)/(PPmax + PPmin)/2]. We compared the accuracy of VRV and PPV for separation of fluid responders and fluid non-responders using receiver operating characteristic (ROC) curves. VRV was better (AUC of the ROC at 0.96, 95% confidence interval 0.92–0.98) than PPV (AUC of the ROC at 0.85, 95% confidence interval 0.79–0.89) for identification of fluid responders, as described in Fig. 5. Differences between VRV and PPV can also be seen in Fig. 6, which shows a Bland–Altman plot of agreements in measurements between VRV and PPV. It shows the biggest difference between VRV and PPV is around the VRV cutoff point for fluid responsiveness.

### Comparison of VRV index and static hemodynamic markers

**Fig. 7:**
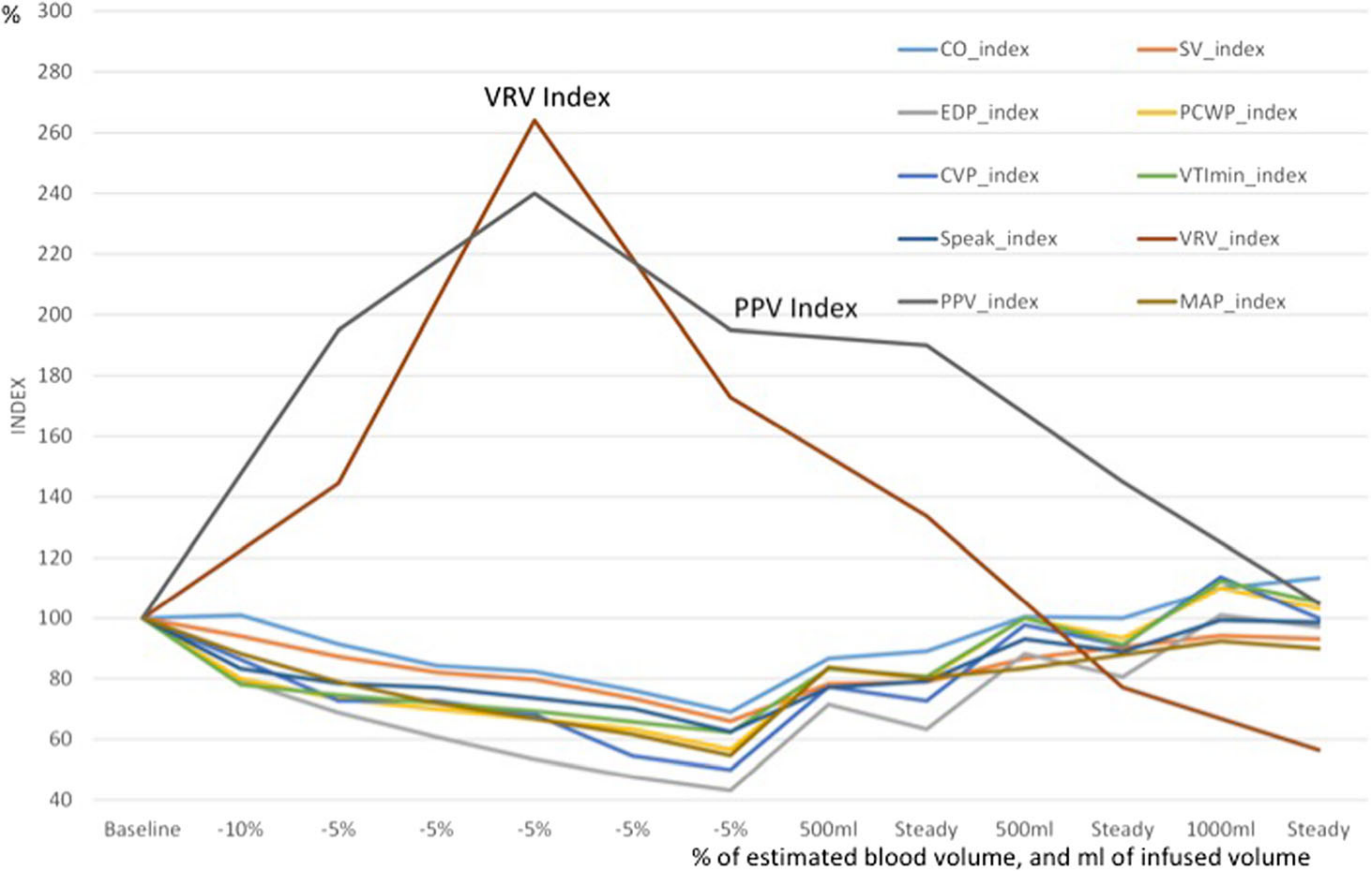
Shows changes in the VRV index, VTI_min_ in SVC, and static markers for hypovolemia. Values are normalized to 100 points at the baseline. This means that all variables started at 100%, and the graphs show their changes during the different stages of the experiment. VRV venous return variation index, PPV pulse pressure variations, CO cardiac output, EDP end-diastolic pressure, CVP central venous pressure, S peak systolic peak velocity, SV stroke volume, PCW pulmonary capillary wedge pressure, VTI velocity time integral, MAP mean arterial pressure

The correlation of the dynamic markers of volume status (VRV index and PPV index) to static markers of hypovolemia—with normalized values to 100 points at the baseline—is presented in Fig. 7. This figure shows relative changes from the baseline. The VRV index exhibited the highest relative change during both hypovolemia and hypervolemia.

## Discussion

This experimental study was performed to validate a new marker for hemodynamic status—the Venous return variation index (VRV). This index is a venous analogy of the arterial SVV index. It is generated by monitoring the respiratory blood flow variations where they occur in the SVC. Performing measurements in the SVC allows for evaluation of changes in right ventricle filling caused by lung–heart interactions, thereby eliminating many factors leading to false measurements (both positive and negative) associated with indexes acquired on the arterial side. The VRV can be easily calculated from changes in blood flow velocity in the superior vena cava (using VTI of flow) during mechanical ventilation. The VRV index closely reflects changes in right ventricular preload. Its measurement is easy, using a Doppler wire introduced to the superior vena cava via a central venous catheter. A Doppler sensor, on a regularly sized guidewire, can easily be inserted through a catheter’s distal lumen. The Doppler signal of the SVC blood flow appears when the sensor exits the catheter’s distal opening. Successful monitoring depends on the catheter tip being positioned within the laminar flow zone in the SVC. During central venous catheter placement, a Doppler sensor can also help to avoid a dangerous wall or near-wall catheter tip placement position. Unintended right atrial placement can also be easily detected from its unique waveform. We have found the Doppler signal to be very stable over the course of many hours in individual cases. The catheter tip had to be placed within a solid column of the laminar flow zone (at least 1 cm long).

We observed interesting changes in both the VRV and the PPV indexes during the bleeding phase of the study. The steepest changes were seen during the first four bleeding stages. Further bleeding did not result in any increase for these indexes. Rather, a decrease was observed in both indexes. This reaction seems paradoxical at first. However, the situation is quite complex. The VRV index is defined as the ratio of VTI_min_ during inspiration and VTI_max_ during expiration. VTI_min_ steeply decreases during the early bleeding stage, while VTI_max_ does not change as much. Further bleeding leads to a more significant decrease of VTI_max_ than VTI_min_ resulting in a decrease of the VRV index during more significant bleeding. However, the VRV index remains above the threshold for hypovolemia. It is important to mention that the absolute value of VTI_min_ continues to decrease during bleeding and can help distinguish normalization of the VRV index during volume therapy from a decrease of the VRV index during prolonged bleeding.

This type of behavior was also seen with PPV. Previously published trials found a continuous decrease of PPV during bleeding [19, 20]. Unlike these studies, we performed milder bleeding. After an initial loss of 10% of estimated total blood volume, further bleeding steps were performed with 5% of estimated TBV, separated by stabilization periods lasting 10 min. This milder bleeding allows activation of compensation mechanisms. Vasoconstriction increases vessel stiffness and preload from the venous reservoir. Increased preload is also partially responsible for the decrease in VRV during later bleeding periods.

The VRV index can be used for continuous measurement to detect changes of hemodynamic status and for the guidance of volume therapy. Currently recommended dynamic indices for hemodynamic monitoring (pulse pressure variation, systolic pressure variations, or echocardiography-based SVV) still have many limitations.

Types of ventilation and cardiac arrhythmias can cause misinterpretations of the respiratory variations in arterial blood pressure [20]. Other causes of misinterpretation are small variations in intrathoracic pressure during ventilation with low tidal volumes (less than 8 ml/kg [21]), lung compliance less than 30 ml/cm H_2_O [22], high frequency ventilation [23], increased intra-abdominal pressure [24], and open-chest surgery [25]. Further limitations of left pressure-based techniques are changes of vessel tone caused either by treatment or by a patient’s condition (for example sepsis). Vasoconstriction decreases—and vasodilatation increases—respiratory-induced changes in blood pressure without any relationship to cardiac output [26, 27].

The VRV index has not yet been tested during these conditions. This is the work of future studies. We tested the VRV index only during mechanical ventilation without spontaneous breathing, and none of the experimental animals had any significant arrhythmias. Anecdotally, we observed the behavior of the VRV index and PPV during ventilation with low tidal volume (5 ml/kg) in both experimental animals and in several clinical cases in patients with hypovolemia. The VRV index stayed in the responsive zone (above 33), while PPV frequently dropped into the non-responsive or gray area (below 15).

## Conclusions

The VRV index can be a fully automated, fast, real-time method, developed for continuous usage with the most pronounced changes evident during early stages of hypovolemia or fluid overload. This method combines the advantages of echocardiography-based methods with a direct and continuous assessment of right ventricle filling during intrathoracic pressure fluctuations caused by mechanical ventilation. Current European guidelines, published in 2017, emphasize the U-shaped curve relationship between hypovolemia and volume replacement. Infusing too much can be as deleterious as infusing too little. Artificial hypervolaemia predisposes patients to interstitial edema, which appears to be associated with perioperative mortality. They also prioritize dynamic parameters, such as SVV or PPV, over static ones and mention Doppler-based devices for assessment of cardiac preload. Our approach follows all these recommendations. We aimed to improve detection of the early phase of hypo- and hypervolemia [28].

### Study limitations

The main limitations of this study were the small number of animals and the use of only mechanical ventilation in fully sedated animals. However, we analyzed 44 hemodynamic situations to test the VRV index as a predictor of fluid responsiveness. Mechanical ventilation was chosen to analyze the VRV index during precisely defined conditions that can be repeated at any time. We used blood at room temperature for blood transfusions. This could influence the cardiac output measurement due to thermodilution. We were able to minimize this influence to a negligible level.

All measurements were understood and analyzed as independent values. Multi-level analysis was not possible due to the low number of experimental animals. Additionally, we only measured flow in the SVC, as this approach most accurately reflects routine clinical practice.

## Abbreviations

AP: Arterial pressure
CCO: Continuous cardiac output
CVP: Central venous pressure
LV: Left ventricle
PAP: Pulmonary arterial pressure
PCw: Pulmonary capillary wedge pressure
PPV: Pulse pressure variation
ROC: Receiver operating characteristic curve
RSVT: Respiratory systolic variation test
RV: Right ventricle
S peak: Systolic peak velocity
SPV: Systolic pressure variation
SVC: Superior vena cava
SvO_2_: Mixed venous saturation
VR: Venous return
VRV: Venous return variation index
VTI: Velocity time integral
